# An Analysis of the Relationship Between the Heat Index and Arrivals in the Emergency Department

**DOI:** 10.1371/currents.dis.64546103ed4fa0bc7c5b779dd16f5358

**Published:** 2015-10-29

**Authors:** Matthew Levy, Morgan Broccoli, Gai Cole, J Lee Jenkins, Eili Y. Klein

**Affiliations:** Department of Emergency Medicine, Johns Hopkins University, Baltimore, MD, USA; Johns Hopkins University School of Medicine, Baltimore, MD, USA; Department of Emergency Medicine, Johns Hopkins University, Baltimore, MD, USA; Department of Emergency Medicine, Johns Hopkins University, Baltimore, MD, USA; Department of Emergency Medicine, Johns Hopkins University, Baltimore, MD, USA; Center for Disease Dynamics, Economics, and Policy, Washington, DC

## Abstract

Background: Heatwaves are one of the most deadly weather-related events in the United States and account for more deaths annually than hurricanes, tornadoes, floods, and earthquakes combined. However, there are few statistically rigorous studies of the effect of heatwaves on emergency department (ED) arrivals. A better understanding of this relationship can help hospitals plan better and provide better care for patients during these types of events.

Methods: A retrospective review of all ED patient arrivals that occurred from April 15 through August 15 for the years 2008 through 2013 was performed. Daily patient arrival data were combined with weather data (temperature and humidity) to examine the potential relationships between the heat index and ED arrivals as well as the length of time patients spend in the ED using generalized additive models. In particular the effect the 2012 heat wave that swept across the United States, and which was hypothesized to increase arrivals was examined.

Results: While there was no relationship found between the heat index and arrivals on a single day, a non-linear relationship was found between the mean three-day heat index and the number of daily arrivals. As the mean three-day heat index initially increased, the number of arrivals significantly declined. However, as the heat index continued to increase, the number of arrivals increased. It was estimated that there was approximately a 2% increase in arrivals when the mean heat index for three days approached 100°F. This relationship was strongest for adults aged 18-64, as well as for patients arriving with lower acuity. Additionally, a positive relationship was noted between the mean three-day heat index and the length of stay (LOS) for patients in the ED, but no relationship was found for the time from which a patient was first seen to when a disposition decision was made. No significant relationship was found for the effect of the 2012 heat wave on ED arrivals, though it did have an effect on patient LOS.

Conclusion: A single hot day has only a limited effect on ED arrivals, but continued hot weather has a cumulative effect. When the heat index is high (~90°F) for a number of days in a row, this curtails peoples activities, but if the heat index is very hot (~100°F) this likely results in an exacerbation of underlying conditions as well as heat-related events that drives an increase in ED arrivals. Periods of high heat also affects the length of stay of patients either by complicating care or by making it more difficult to discharge patients.

## Introduction

Heatwaves are one of the most deadly weather-related phenomenon in the United States and account for more deaths annually than hurricanes, tornadoes, floods, and earthquakes combined^1^. The National Weather Service (NWS) defines a heat wave as “a period of abnormally and uncomfortably hot and unusually humid weather”, typically lasting two or more days^2^. As climate change increases the mean and variance of average temperatures, the incidence and severity of heatwaves are expected to continue to increase^3^.

While heatwaves have significant consequences on population health, they can also stress the health system, especially emergency departments. A number of studies have suggested that heatwaves significantly increase emergency department (ED) visits and workload^4^^,^ 5 ^,^ 6 ^,^ 7 ^,^ 8 ^,^ 9 ^,^ 10. The effect of heatwaves is multifactorial and is based on the extent of the temperature increase, the region’s climate, the socio-economic background of the population, and the preparedness and resiliency of the population 6 . In addition, determining the effect of a heatwave on ED arrivals can be difficult as comparing a heatwave to different times in the same summer or to just a single other year can bias the results due to other factors such as a general upward trend in ED usage^11^ or the timing of the heatwave (e.g., a heatwave over a weekend or holiday may limit other care options). There are a limited number of statistically rigorous analyses of the effect of heatwaves on emergency department utilization in temperate US cities. A better understanding of this information is essential for both governmental and hospital emergency planners to develop heatwave action plans that address the surge capacity of emergency departments.

On June 29th, 2012, health officials in Maryland declared a statewide heat emergency. In accordance with Maryland’s Heat Emergency Plan, a heat emergency represents the state’s most severe type of heat event^12^. Multiple factors can trigger the declaration of a heat emergency. These factors include, but are not limited to: Critical infrastructure damage in the form of significant power or water outage; periods of extended heat waves lasting more than three days; or other factors that would exacerbate a heat emergency. During the time of this heat emergency, several temperature records were set, with individual highs as severe as 104°F.^13^^,^ 14 This heatwave helped make July 2012 the hottest month on record for the contiguous United States^15^. The aim of this study was to examine the effect of heatwaves in the context of annual summer temperatures. Specifically, we examined whether this particular heat emergency increased ED arrivals, and whether a general relationship between heatwaves and ED arrivals existed. We examined these effects in Baltimore, which has a temperate climate representative of large swaths of the US, making it a useful city to investigate the effects of extreme heat on emergency department arrivals.

## Methods

**Study design and setting**

A deidentified retrospective review of all ED patient arrivals for the years 2008 through 2013 including data only from April 15 through August 15 for each year was performed. The purpose was to explore and describe the interaction of daily/hourly number of ED arrivals and heat related weather variables. The research team sought to assess a relationship between the heat index and ED arrivals during a heat wave with the hypothesis being a positive association between the two. This study was performed in the adult and pediatric ED of The Johns Hopkins Hospital (JHH) in Baltimore, Maryland, USA, which serves over 104,000 patients annually. While these are two geographically adjoining facilities, they were considered one ED in this analysis.

**Selection of Participants**

Deidentified daily and hourly patient arrival data were extracted retrospectively from electronic logs for ED visits during the 5 year study period. The number of arrivals was aggregated on both an hourly and daily basis and stratified by chief complaint, patient acuity, and discharge diagnosis codes. Inclusion criteria were all patients who presented to the ED during the study period. No patients were excluded during the study period. The study was determined to be not human subjects research by the Johns Hopkins School of Medicine Institutional Review Board.

**Analysis**

The NWS uses the heat index (HI), which includes the effects of ambient air temperature and relative humidity, rather than absolute air temperature, to trigger heat warnings, as the HI is a more accurate representation of the human body’s perception of temperature. The heat index was calculated using hourly measures of temperature and relative humidity obtained from an online database (http://www.wunderground.com) for the five digit zip code of Johns Hopkins Hospital (see Appendix for heat index calculation).

Scatter plots were created to illustrate the overall relationship (linear, curvilinear, etc.) between the heat index and the number of arrivals on an hourly and daily basis. Locally-weighted regression (LOWESS)^16^^,^ 17 was used to provide a smoothed fit to the data. To avoid autocorrelation, generalized additive models (GAMs)^18^^,^ 19 ^,^ 20, were used to further examine the relationships between the variables. GAMs have been effectively applied in numerous biological fields, including genetics, epidemiology, molecular biology, and medicine^21^. They are also the most widely applied method in studies of air pollution and mortality^21^. GAMs were chosen because the method allows for nonparametric adjustments for non-linear confounding effects, and other common methods, such as Box-Jenkins and Poisson models have shown similar results^9^. The following GAM was fit,

\begin{equation*}{Y_t}\~Poisson({\mu _t})\\\%0A\log {\mu _t} = \alpha  + {f_1}(tim{e_t}) + {f_2}(H{I_t}) + facilit{y_t} + weeken{d_t} + heatwav{e_t}\end{equation*}

where *Y_t_* is the number of daily arrivals, *HI_t_* is the maximum daily heat index value, *time_t_* is the date, and *weekend_t_* is a binary variable. In May 2012, the ED moved to a new building, so a binary variable denoting this was added, *facility_t_*. We also analyzed the effect of the 2012 heatwave (June 29, 2012-July 13, 2013) using a binary variable, *heatwave_t_*. Sub-analyses of arrivals by age and acuity were also performed. We examined the effect of the heat index on a single day as well as the cumulative effect of the heat index over three days (the current day plus the prior two preceding days). A P value ≤ 0.05 was considered statistically significant, determined by anova.

Because ED operations are effected by the amount of time patients spend in the department (i.e., length of stay [LOS]), we estimated a second model that examines the effect of the heat index on the length of time patients spent in the emergency department controlling for the number of daily arrivals. The second GAM model fit was,

\begin{equation*}{L_t}\~Poisson({\mu _t})\\\%0A\log {\mu _t} = \alpha  + {f_1}(tim{e_t}) + {f_2}(H{I_t}) + {f_3}({Y_t}) + facilit{y_t} + weeken{d_t} + heatwav{e_t}\end{equation*}

where *L_t_* is the mean length of stay in minutes for patients on each day. Finally, we also looked at the effect of the heat index on dwell time for patients discharged from the ED, defined as the time a patient was placed in an exam room to when a disposition decision was made and recorded by the provider. For robustness, the relationship between LOS/dwell time and the heat index was also estimated using a negative binomial regression. Analyses were done in R 3.0.2^22^ using the locfit^23^ and mgcv^24^^,^ 25 packages, and in Stata 12.1 (StataCorp LP).

## Results

During the 5 year study period there were a total of 91,578 ED patient arrivals from 372 24-hour periods spanning June 15 through August 15. The average number of daily arrivals over the course of the study was 246 (SD: 27). Coinciding with the transition to new facilities in April 2012, the average daily arrival of 237 (SD: 23) patients per day increased to 265 (SD: 25) patients per day (Figure 1 shows a box-whisker diagram describing average arrivals by year). Over the period of study, the heat index ranged from 65.4 to 116.8 (Figure 2 shows the histogram of maximum daily heat index), and a non-uniform association between the maximum daily heat index and patient arrivals on that day was observed using LOWESS. Figure 3 displays the observed relationship between the heat index and the number of arrivals which was characterized by a slight decrease followed by an increase as the heat index rose and then a slight decrease at very high levels.

**Figure:**
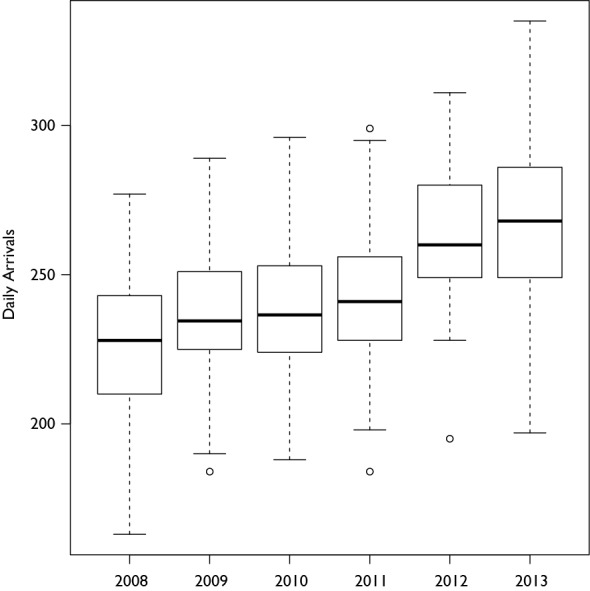
**Figure 1: Boxplot of Arrivals per Day by Year.** The data is the number of daily arrivals from June 15 through August 15 for each year. The number of average daily arrivals remained relatively steady from 2008 to 2011, but after the new Johns Hopkins Hospital was opened in April 2012, there was an increase in the average number of daily arrivals.

**Figure:**
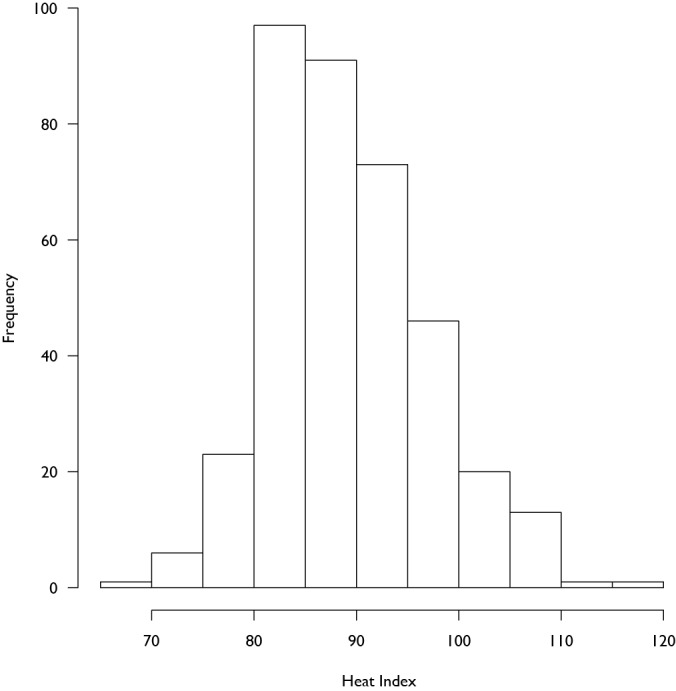
**Figure 2: Histogram of Daily Maximum Heat Index. **Distribution of the daily heat index over the study period.

**Figure:**
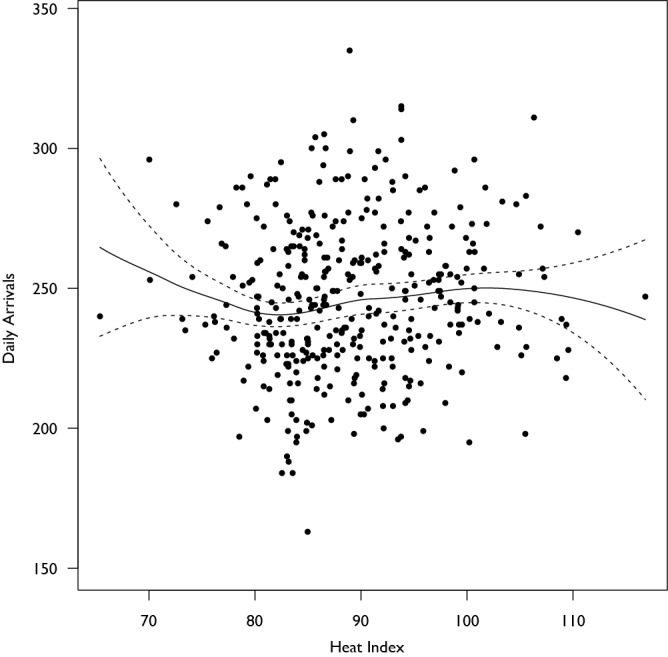
**Figure 3: Scatterplot of arrivals per day and the Heat Index for the Day. **The scatterplot shows no distinct trend on observation, though analysis with locally-weighted regression (LOWESS) suggests that there may be some non-uniformity to the relationship between the heat index and the number of arrivals, with an increase at higher heat index ranges.

No effect of the heat index of a single day on the number of arrivals was observed, but a cumulative effect over multiple days was observed to significantly affect arrival patterns. Figure 4 summarizes the result of the effect of the mean heat index of the current plus two preceding days on the number of arrivals in the emergency department. As expected the weekend variable was negative and significant (p<.001), reflecting fewer arrivals to the emergency department on weekends, and the facility variable was positive and significant (p<.001), reflecting the observed fact that arrivals increased when the new facility opened. The heat index (p=.02) and time (p<.001) were significantly associated with arrivals, and the effect of the heat index was non-linear, with an increasing number of arrivals, or about a 2% increase in arrivals, when the mean heat index for three days approached 100°F. However, the heat wave variable (for the extended hot period in July 2012) was not significantly associated with arrivals after controlling for the heat index. The same analysis using the mean maximum temperature over three days found similar results, but temperature was not as good a predictor.

**Figure:**
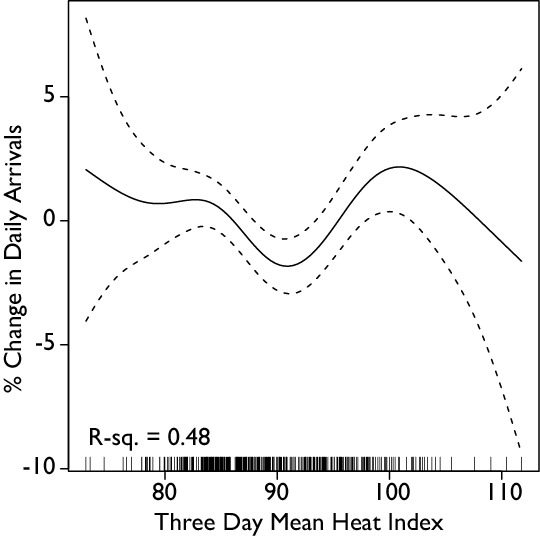
**Figure 4: Generalized Additive Model Analysis of Mean Three Day Heat Index on Daily** **Arrivals.** The mean three day heat index is the mean of the heat index for the day in question and the two preceding days. The data shows a pronounced and significant decrease in admissions as the mean three-day heat index increases, but then significantly increases again as the mean three-day heat index increases further. This suggests that when it gets hotter, but not too hot, people restrict their activities, which results in fewer emergency department visits. However, when it has been hot for a couple days in a row, this leads to exacerbations of underlying problems and more visits to the emergency department.

An analysis of the effect of the heat index on arrivals by age found that the heat index produced non-linear patterns of arrivals for children and adults aged 18-64, but no significant pattern for the elderly (65+). Precision in the child and adult arrivals was diminished, not reaching significance in the children and was only marginally significant in the adults (p=.08). Again, the heat wave variable was not significantly associated with arrivals at any age level. Figure 5 summarizes the relationships for children, adults, and the elderly. Further analysis by level of acuity, found that acute arrivals (emergency severity index (ESI) levels 1 and 2 [40]) were not significantly affected by the heat index, but that non-acute patients (ESI levels 3-5) had a similar significant pattern as above (p=.03). These different patterns are shown in Figure 6.

**Figure:**
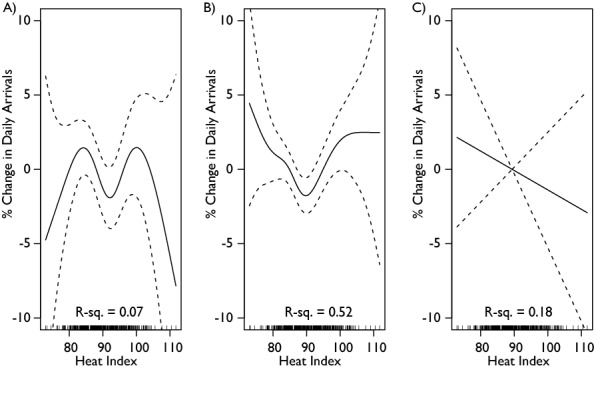
**Figure 5: Generalized Additive Model Analysis of Mean Three Day Heat Index on Daily** **Arrivals by Age.** The heat index is the mean of the heat index for the day in question and the two preceding days. A is children (0-17), B is adults (18-64), and C is elderly (65+). The pattern for adults and children is similar to the overall picture (Figure 3), but interestingly, increasing heat seems to have little effect on the elderly.

**Figure:**
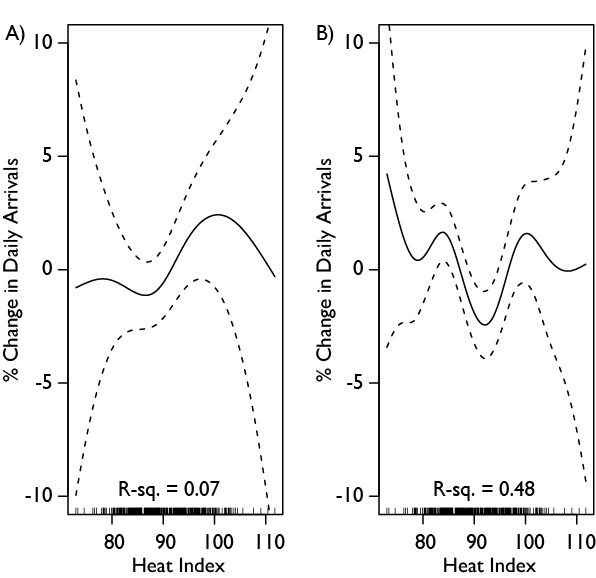
**Figure 6: Generalized Additive Model Analysis of Mean Three Day Heat Index on Daily** **Arrivals by Acuity.** The heat index is the mean of the heat index for the day in question and the two preceding days. A is patients with an acuity level of 1 or 2 (higher acuity), while B is patients with acuity levels 3-5 (lower acuity). The overall pattern is thus driven by a combination of increasing arrivals of sicker patients as the heat index increases, as well as those with lower acuity.

Finally, we found that an increase in the heat index resulted in an increase in the length of stay for all patients of approximately 5% when the mean heat index for three days exceeded 100°F (Figure 7). In this case however, we found that the heat wave variable was significant for length of stay (p<.001). For length of stay, the heat wave was correlated with a ~10% increase in the LOS. The average LOS during the heat wave was 514 minutes compared with 423 minutes for non-heat wave days. A Students t-test finds these means to be significantly different (p<.001). These results were qualitatively the same when estimated using a negative binomial regression. No statistically significant effect was found on the effect of the heat index or 2012 heatwave on the dwell time of the patient.

**Figure:**
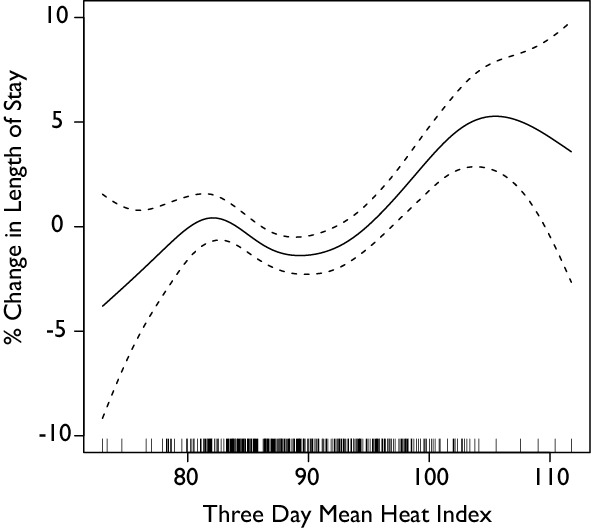
**Figure 7: Generalized Additive Model Analysis of Mean Three Day Heat Index on Length of Stay. **The heat index is the mean of the heat index for the day in question and the two preceding days

## Discussion

Analysis of this study’s dataset revealed a statistically significant association between a sustained elevated heat index over a three day period and increased ED volume of lower acuity patients. However, the pattern of arrivals related to changes in the heat index was nonlinear and appeared to decrease as the heat index increased and then increase again as the heat index approached 100°F. Surprisingly, in contrast to other studies, we found no significant effect of the 2012 heat wave on ED arrivals. This could be due to the fact that a heat emergency was declared, which instituted plans that helped mitigate risk to individuals of heat-related complications. Alternatively, though it was hot for a sustained period of time during that particular heat wave, there were other periods of time over the years that approached or had similar heat index values, and it is not just extended periods of high-heat for weeks that can cause heat-related trips to the ED, but shorter time-periods of only a few days.

The findings of this study help to explain the common subjective perception that ED volume seems higher or that it is busier during days with increased heat. The problem isn’t exclusively one of arrivals, but also one of length of stay; i.e., it took longer to move patients out of the ED during these times. This is seen in the increased overall mean length of stay for all patients. While the percentage of patients admitted wasn’t higher, the increased arrivals may have contributed to an overall busier ED, which made it harder to move patients through and in some cases discharge patients home as demand for a fixed set of resources increased.

Alternatively, the increased heat contributes to longer lengths of stay either by complicating care or by making it more difficult to discharge patients. The latter is a strong possibility, as the urban, lower socioeconomic status, and homeless populations served by this study’s ED are at increased risk of heat-related mortality^26^^,^ 27 ^,^ 28. In a patient population where home air conditioning is not ubiquitous^27^, which is a significant risk-factor for heat-related illness^27^^,^ 29 ^,^ 30, discharging those in ‘at risk’ populations (e.g. chronically ill, drug/alcohol dependency, obese, homeless) increases complexity and risk. Such cases frequently require additional time, resources, and assistance as part of the discharge planning process to ensure safe patient outcomes.

The increase in ED arrivals as the heat index increased is similar to prior research that found heatwaves significantly increase ED visits.^4^^,^ 5 ^,^ 6 ^,^ 7 ^,^ 8 ^,^ 9 ^,^ 10 Most other studies only used methods that could detect linear changes in effects of heatwaves due to changes in temperature or examined effects above a threshold level.^35^^,^ 36 However the non-linearity of our result is similar to results from Madrid, Spain^9^ which showed a general decrease in the number of arrivals as the temperature increased before significantly increasing when the temperature rose above 36 °C. More recent studies in New York^36^ and Hong Kong^35^ also found non-linearities in the effect of temperature and humidity on hospital admissions. Since the heat index and temperature are highly correlated (Figure 8), we expect that this non-linearity may be somewhat common in the nature of arrivals to EDs as a consequence of increasing temperature, though more analysis is necessary.

**Figure:**
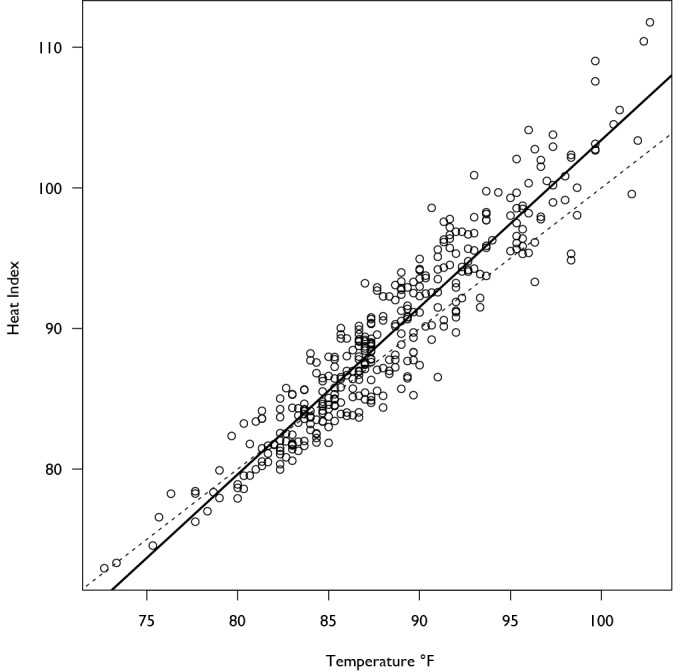
**Figure 8: Scatterplot of Temperature and Heat Index. **The solid line is the linear regression of the heat index on temperature and the dashed line has a slope of 1, showing that the heat index increases at a slightly faster rate than the temperature.

In this series, we observed a relationship between a sustained high heat index and ED arrivals over a 72hr period, suggesting heat waves effects are cumulative in nature. The longer the environment stays hot, the more likely it is that existing medical conditions will be aggravated. Given that such events tend to span several operational periods, and that cumulative effects manifest more apparently after 3 day increments (thus providing some planning time), opportunities exist to adjust staffing configurations and resource availability. It has been suggested elsewhere that clinical staffing during heatwaves be increased^31^^,^ 32. However, given financial realities, a more thoughtful approach might be to have incremental staff on standby or on call to either come to the ED or support it via a telemedicine infrastructure. Examples of such incremental ED clinical staff worthy of consideration include case managers and social workers who can provide heat-related behavioral advice to patients^33^ and ensure appropriate post-discharge care coordination.

An alternative to this approach, especially for EDs lacking 24-hour case management or social work coverage, is to provide care coordination education to the clinical staff so that they can affect the connection between patients and appropriate external resources. This goes beyond providing free taxi vouchers or bus tokens to get patients to the nearest shelter or cooling center. To truly create organizational capacity for care coordination, ED staff must understand what resources exist and under what circumstances each is best leveraged to support post-discharge patient needs. EDs may develop standardized criteria or assessment tools to assist their staff in determining what resources are most appropriate for varying patient social circumstances. EDs may also consider incorporating such templates into their electronic documentation systems for subsequent inclusion into patient health records.

**Limitations**

As with any retrospective study there are limitations to the analysis. While we capture environmental variables and arrivals to the ED, the results are correlative and the exact causes that drove people to the emergency department are not examined or known. This may bias the results somewhat downward, as one would expect that heat-related arrivals would be more common as the heat index increased. A second limitation is that while this study took place in a temperate US city that is representative of many areas of the US, it is a single center that may have different norms or cultural factors that could limit its generality. In addition, though other cities may have a similar environment and climate, minor climatic differences may be magnified in this research.

## Conclusion

This study found that when the heat index stayed high for several days in a row (regardless of a declared heat emergency) the ED saw an increased volume of lower acuity patients. However, this pattern was non-linear, first falling as the heat index increased and then increasing again, a pattern not previously described. Moreover, our results demonstrate that increased heat also increases patient LOS, which together with increased arrivals increases crowding in the ED. Operational considerations as a result of this data may include increasing just in time staffing given the extended clinical nature of the events and case management and social work staffing for disposition planning.

## Appendix

The maximum daily heat index was calculated using the following equation:

$\begin{array}{c}\%0AHeatIndex =  - 42.379 + 2.04901523T + 10.14333127R - .00683783{T^2}\\\%0A - 0.05481717{R^2} + 0.00122874{T^2}R + 0.00085282T{R^2} - 0.00000{\rm{199}}{{T}^2}{R^2}\%0A\end{array}$

where T is the ambient temperature in degrees Fahrenheit and R is the percent relative humidity^34^. Because this equation was obtained by regression analysis, an adjustment is required for certain temperatures and humidity. The following adjustment is subtracted from the heat index when the relative humidity is less than 13% and the temperature is between 80 and 112 degrees Fahrenheit,

\begin{equation*}{HI - \frac{{13 - R}}{4}\sqrt {\frac{{17 - \left| {T - 95} \right|}}{{17}}} }\end{equation*}

The following adjustment is added to the heat index if the relative humidity is greater than 85% and the temperature is between 80 and 87 degrees Fahrenheit,

\begin{equation*}{HI + \frac{{R - 85}}{{10}}\frac{{87 - T}}{5}}.\end{equation*}

## Competing Interests

The authors have declared that no competing interests exist.
